# Structural Rearrangement of Dps-DNA Complex Caused by Divalent Mg and Fe Cations

**DOI:** 10.3390/ijms22116056

**Published:** 2021-06-03

**Authors:** Liubov Dadinova, Roman Kamyshinsky, Yury Chesnokov, Andrey Mozhaev, Vladimir Matveev, Andrey Gruzinov, Alexander Vasiliev, Eleonora Shtykova

**Affiliations:** 1 Shubnikov Institute of Crystallography of Federal Scientific Research Centre “Crystallography and Photonics” of Russian Academy of Sciences”, Leninskiy Prospect, 59, 119333 Moscow, Russia; kamyshinsky.roman@gmail.com (R.K.); chessyura@yandex.ru (Y.C.); a.a.mozhaev@gmail.com (A.M.); a.vasiliev56@gmail.com (A.V.); eleonora.shtykova@gmail.com (E.S.); 2 National Research Center “Kurchatov Institute”, Akademika Kurchatova, 1, 123182 Moscow, Russia; 3 Moscow Institute of Physics and Technology, Institutsky Lane 9, 141700 Dolgoprudny, Russia; 4 Shemyakin-Ovchinnikov Institute of Bioorganic Chemistry, Russian Academy of Sciences, Miklukho-Maklaya, 16/10, 117997 Moscow, Russia; 5 Physics Department, Lomonosov Moscow State University, 119991 Moscow, Russia; matveeff.volodia2012@yandex.ru; 6 EMBL, Hamburg Outstation, c/o DESY, Notkestr. 85, Geb. 25a, 22607 Hamburg, Germany; agruzinov@embl-hamburg.de

**Keywords:** DNA–Dps co-crystals, DNA–protein interaction, small-angle X-scattering, cryo-electron microscopy

## Abstract

Two independent, complementary methods of structural analysis were used to elucidate the effect of divalent magnesium and iron cations on the structure of the protective Dps-DNA complex. Small-angle X-ray scattering (SAXS) and cryo-electron microscopy (cryo-EM) demonstrate that Mg^2+^ ions block the N-terminals of the Dps protein preventing its interaction with DNA. Non-interacting macromolecules of Dps and DNA remain in the solution in this case. The subsequent addition of the chelating agent (EDTA) leads to a complete restoration of the structure of the complex. Different effect was observed when Fe cations were added to the Dps-DNA complex; the presence of Fe^2+^ in solution leads to the total complex destruction and aggregation without possibility of the complex restoration with the chelating agent. Here, we discuss these different responses of the Dps-DNA complex on the presence of additional free metal cations, investigating the structure of the Dps protein with and without cations using SAXS and cryo-EM. Additionally, the single particle analysis of Dps with accumulated iron performed by cryo-EM shows localization of iron nanoparticles inside the Dps cavity next to the acidic (hydrophobic) pore, near three glutamate residues.

## 1. Introduction

It is well known that metal ions modulate various biological processes including interactions of polynucleotides with protein macromolecules, which are of particular importance [1]. Nucleic acids are highly charged polyanionic biopolymers; thus, cations can alter DNA or RNA structures [2] or change their interaction with biological partners [3]. For example, in the process of interaction with polycations (cationic protein and polyamines) DNAs can be tightly packaged inside cell nucleus, bacterial cytoplasm and viral capsids [4,5].

One of the most important, yet insufficiently studied processes is a formation of the protective crystalline complex of DNA with stress-induced protein Dps (DNA Binding Protein from Starved Cells) [6]. It was shown that Dps binds to DNA non-specifically, and the process is mediated mainly by electrostatic interaction [7,8]. Therefore, the presence of divalent metal cations such as Mg^2+^ and Fe^2+^ plays an important role in the formation and co-crystallization of the Dps-DNA complex, depending on the concentration of the cations in the cytoplasm of bacterial cells. During long starvation, the concentration of the ions in the nutrient medium decreases below the threshold value, and electrostatic interaction between negatively charged DNA bases and positively charged amino acids of the Dps dodecamers occurs, leading to well-known co-crystallization in living cells. Conversely, when fresh nutrients containing divalent cations are supplied, the complex is destroyed [8].

The ability of Dps to bind to DNA is determined by the disordered lysine-rich N-termini located on the surface of the dodecamer [9], and members of the Dps family, which do not contain these positively charged regions, cannot bind DNA [10,11,12]. Metal-binding sites located on the external surface of the dodecamer also play the essential role in the self-assembling of the protein and its binding with DNA [13]. For example, N-terminal fragments of DrDps1 from *Deinococcus radiodurans* contain the outer metal-binding site (residues 30–55) with the motif Asp36*x*_2_His39*x*_10_His50*x*_4_Glu55, and its disruption reduces the DNA-binding ability of the protein [14].

In addition to external metal binding sites, Dps family members contain highly conserved inner ferroxidase centers (FOCs) [15], where Fe^2+^ ions are oxidized by hydrogen peroxide to produce Fe^3+^, which is mineralized and stored within the Dps cavity in a bioavailable and non-toxic form [16,17].

Therefore, the Dps protein protects bacterial cells performing dual functions; however, DNA binding and iron oxidation occur completely independently of each other [18]. It means that iron and hydrogen peroxide detoxification properties of the protein may interfere with its ability to bind DNA, and vice versa. Thus, it is important to study the effect of metal cations, especially iron cations, on the ability of a protein to form a protective crystalline complex with DNA.

To study this process in solution, the cations can be added to the solvent to reduce and modulate the repulsion between DNA and Dps. It was shown in [8] that a chelating agent such as EDTA (ethylenediaminetetraacetic acid) also affects the complex formation. Moreover, formation of Dps-DNA co-crystals is influenced not only by the presence of divalent cations and chelating agents, but also by pH and ionic strength of the solution [9].

The threshold values of Mg^2+^ concentrations required for the formation of Dps-DNA complex were determined in [8,9,19]. It was found that the complex is not formed at concentrations of MgCl_2_ less than 2 mM, while higher concentrations of the cation (7.5–10 mM) inhibit Dps-DNA complex formation.

Earlier [20], using small-angle X-ray scattering (SAXS), we showed that 10 mM MgCl_2_ almost completely destroys the Dps-DNA co-crystals, while the form-factor of the protein that exists independently of DNA can be clearly traced in solution. The addition of 10 mM FeSO_4_ leads to the complete destruction of the crystalline complex and a general aggregation. Though many aspects of the Dps-DNA interaction have been characterized already, some important details of this phenomenon need further clarification. To advance in this problem, in the present study we examine the effect of two key metal ions, iron and magnesium cations, on the ability of the Dps protein to form crystal structures with DNA. Importantly, Fe and Mg belong to two different types of metals: iron is a transition metal, while magnesium is a non-transition metal. This largely determines their influence on the formation of the crystalline complex due to effect on the conformation of the N-terminal regions of the Dps protein. Thus, one of the most important tasks of our research is to characterize Dps as a key component of the DNA-protein complex and to determine its structural readiness for the complex formation. Here we demonstrate new structural data from SAXS and cryo-electron microscopy (cryo-EM) of the effect of 20 mM MgCl_2_ and FeSO_4_ on both the Dps protein conformation and co-crystal formation. In particular, we show that Mg^2+^ ions block the N-terminals of the protein from interacting with DNA, while the addition of EDTA leads to reverse co-crystallization of Dps-DNA, and iron is localized inside the Dps cavity next to the acidic (hydrophobic) pore, near three glutamate residues.

## 2. Results and Discussion

### 2.1. Influence of Mg^2+^ on Dps-DNA Co-Crystal

To study the effect of divalent Mg^2+^ cations on the Dps-DNA co-crystals obtained as described previously [21,22], 20 mM MgCl_2_ salt was added to the solution of the formed protective quasi-crystalline Dps-DNA complex.

**Figure 1:**
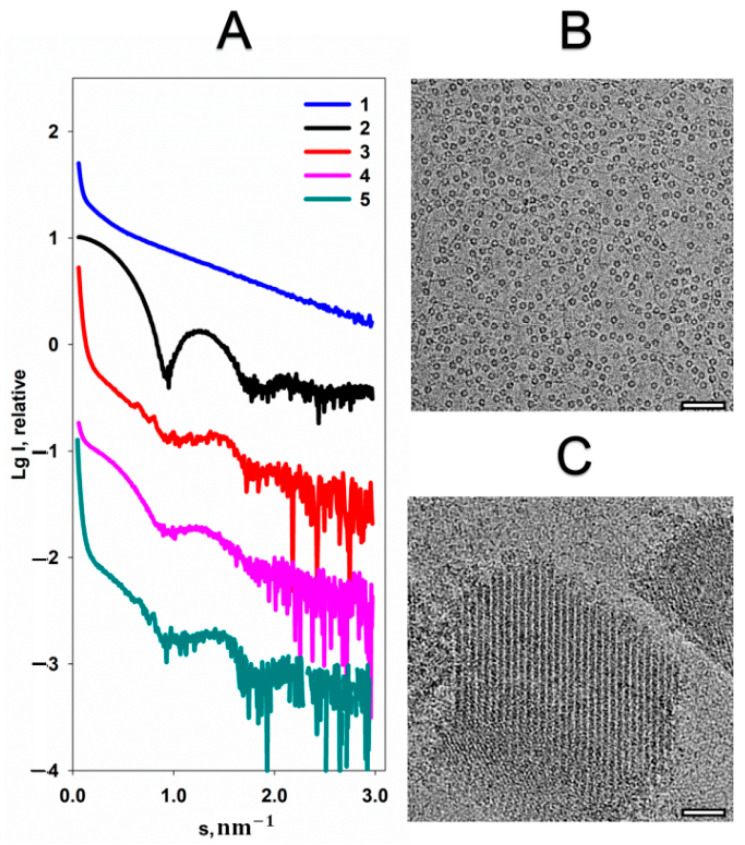
The effect of MgCl_2_ on the structure of Dps-DNA co-crystals. (**A**) Experimental SAXS curves from: 1—DNA, 2—Dps, 3—Dps-DNA complex, 4—Dps-DNA co-crystals with addition of 20 mM MgCl_2_, 5—Dps-DNA co-crystals after addition of 20 mM MgCl_2_ and 20 mM EDTA. Cryo-EM data of Dps-DNA co-crystals after addition of 20 mM MgCl_2_ (**B**) and Dps-DNA co-crystals after addition of 20 mM MgCl_2_ and 20 mM EDTA (**C**). Bar length is 50 nm.

Formation of the quasi-crystalline structure of the Dps-DNA complex is expressed by the appearance of Bragg peaks on the SAXS curve (Figure 1A, curve 3), while the individual components of the complex are characterized only by the scattering from their shapes (form-factors) in solution (Figure 1, curves 1 and 2). Previously, it was demonstrated that the structure of the Dps-DNA complex depends on the buffer composition [21,22]. Since Dps-DNA interaction has mainly electrostatic nature, the addition of any charged component into the solution is expected to affect the structure of the Dps-DNA complex. As seen in Figure 1, addition of 20 mM MgCl_2_ leads to the disappearance of the Bragg peaks, indicating the destruction of the quasi-crystalline structure of the complex (Figure 1A, curve 4), which is fully consistent with previously published data [20]. However, attention is drawn to the clear presence of the scattering from free Dps macromolecules—the form-factor of the protein can be clearly observed on the scattering curve (Figure 1A, curves 2 and 4). It can be assumed, thus, that after the addition of magnesium cations the complex disintegrated into its non-interacting components. To confirm this assumption, the OLIGOMER program [23] was used to quantify the volume fractions of free Dps and DNA in the solution. The calculation demonstrates presence of about 46 vol.% DNA, 54 vol.% Dps and insignificant amounts of their complexes in a solution containing 20 mM MgCl_2_. Therefore, curve 4 in Figure 1A represents just a sum of the scattering from free, non-interacting Dps and DNA. However, the process of the complex disintegration is reversible; an addition of 20 mM EDTA to the solution leads to the reappearance of a quasi-crystalline structure (Figure 1A, curve 5). Hence, we can conclude that the presence of a large number of positive charges (Mg^2+^) in the solution prevents the formation of the complex, closing the negative charges of DNA for interaction with the positively charged amino acids of the protein. Accordingly, when EDTA chelates the magnesium cations, the complex is restored.

A similar picture can be observed from cryo-EM data. Figure 1B shows that upon the addition 20 mM MgCl_2_ to the Dps-DNA solution, protein and DNA particles are distributed evenly throughout the vitreous ice. However, the addition of EDTA leads to the formation of Dps-DNA co-crystals (Figure 1C) having the morphology similar to those described earlier [21].

**Figure 2:**
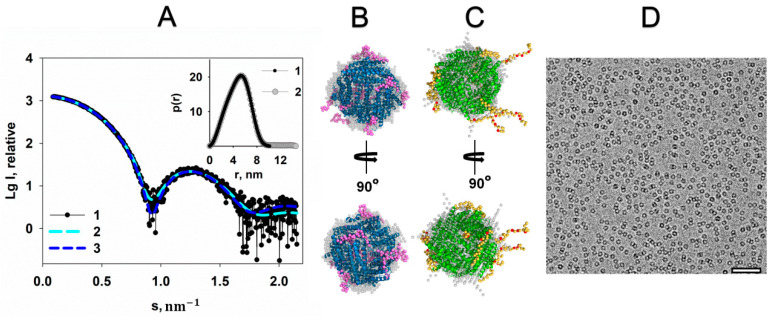
The effect of 20 mM MgCl_2_ on the Dps structure. (**A**) The experimental scattering curve of the Dps protein in the presence of 20 mM MgCl_2_ in solution (1), scattering from DAMMIN (2), and CORAL (3) models of Dps. Insert: the distance distribution function *p(r)* of Dps in the presence of 20 mM MgCl_2_ (1), and Dps without MgCl_2_ (2). (**B**) and (**C**) shows the superposition of the DAMMIN model (gray spheres) and the CORAL in two orientations for better visualization: blue (**B**) and green (**C**) helices—the high-resolution structure of the Dps protein (PDB ID: 1DPS); magenta (**B**) and yellow (**C**) spheres are reconstructed N-terminal regions. (**C**) The residues Lys5, Lys8, Lys10 and Arg18 are represented by red spheres in the CORAL model. (**D**) Cryo-EM data of Dps with MgCl_2_; bar length is 50 nm.

Interactions with negatively charged phosphodiester backbones of DNA occur through positively charged lysine (Lys5, Lys8, and Lys10) and arginine (Arg18) residues located in the N-terminal fragments of Dps from *E. coli* [9,24]. Presence of additional positive charges in solution provided by the MgCl_2_ salt can change the structure of both the N-terminal fragments of the protein and the structure of the whole macromolecule, disrupting its ability to bind DNA. Therefore, evaluation of the effect of MgCl_2_ on the Dps structure was a necessary next step in the present study. Figure 2A shows the scattering curve from the Dps protein in solution containing 20 mM MgCl_2_.

The distance distribution function *p(r)* calculated using the GNOM program [25] from the experimental scattering curve (Figure 2A, insert, curve 1) shows that the maximum size of the protein is about 10 nm. This value correlates well with the size of the protein in the crystal, equal to ~9 nm (PDB ID: 1DPS). It means that the N-terminal regions are mainly pressed against the surface of the protein. *Ab initio* reconstruction of the low-resolution Dps shape using the DAMMIN program [26] shown in gray spheres in Figure 2B supports the conclusion. Since the protein exists in solution as a dodecamer, the symmetry P23 was applied. The discrepancy *χ^2^* = 1.03 indicates good agreement between the experimental curve and the calculated scattering from the low-resolution shapes obtained (Figure 2A, curves 1 and 2).

To determine a more detailed full-length structure of the protein and a configuration of its N-terminal fragments in solution in the presence of Mg cations, the hybrid approach implemented in the CORAL program [27] was employed. The full-length structure of the protein was modeled using atomic coordinates of the available high resolution Dps structure (PDB ID: 1DPS), where the first N-terminal 22 amino acid residues of the protein missing in the crystal lattice were reconstructed by *ab initio* procedure. The model obtained, displayed in Figure 2B, shows that flexible N-terminal regions of the proteins are located close to the protein surface, which makes them inaccessible for interaction with DNA. Model obtained yields a good fit to the experimental data, with *χ^2^* = 1.11 (Figure 2A, curves 1 and 3).

Superposition of structures obtained by two independent methods demonstrates their identity with spatial deviation NSD = 0.94 (Figure 2B).

However, it is necessary to compare the configuration of the N-termini of the Dps macromolecule in the absence of the Mg cations with the results described above. To exclude presence of the cations, which can affect the conformation of the N-terminal regions, the chelating agent EDTA was added to the solution. Distance distribution function *p(r)* for the sample of Dps that does not contain additional metal cations demonstrates an increase of the maximum size *D_max_* of the protein up to 14 nm (Figure 2A, insert, curve 2). Both ab initio and hybrid reconstructions displayed in Figure 2C show that flexible N-terminal regions of the protein freely extended into solution. As one can see from Figure 2C, positively charged Lys5, Lys8, Lys10 and Arg18 residues, which are responsible for the interaction with negatively charged bps of DNA and represented by red spheres in the CORAL model, are quite accessible for the interaction with the polynucleotide, which is in contrast to the previous case, where the N-termini of Dps are located closer to the surface of the protein. The morphology of these Dps regions at the presence of EDTA is similar to that described earlier [28], and indicates the readiness of the protein to interact with DNA.

In turn, the cryo-EM image (Figure 2D) demonstrates evenly distributed Dps particles in solution containing Mg cations without aggregation and other visible differences compared to images of Dps solution without MgCl_2_.

### 2.2. Influence of FeSO_4_ on the Dps-DNA Co-Crystals

Since the ferroxidase activity and iron storage function of the Dps are extremely important for bacterial survival, a study of the influence of the divalent Fe cations on the structure of the protective Dps-DNA complex is of special interest. Unique metal-binding sites locate both on the external surface of the dodecamer and within the hollow cavity in the middle of the Dps dodecamer. These metal-binding sites of ferritin-like proteins, as a rule, are rich in Asp, His and Glu amino acids [13,14,24,29,30,31]; however, the sites are not highly conserved and the amino acids within them can be replaced by Lys, Ala and others.

**Figure 3:**
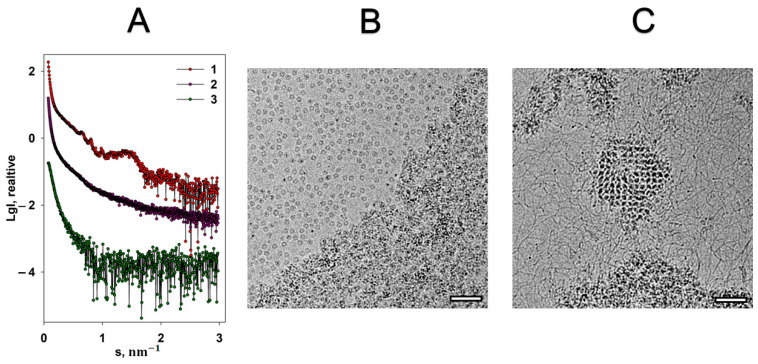
The effect of FeSO_4_ on the Dps-DNA co-crystals. (**A**) Experimental SAXS curves from: 1—Dps-DNA co-crystals, 2—Dps-DNA co-crystals in the presence of 20 mM FeSO_4_, 3—Dps-DNA co-crystals with 20 mM FeSO_4_ and EDTA. Cryo-EM data of Dps-DNA co-crystals with FeSO_4_ (**B**) and Dps-DNA co-crystals with FeSO_4_ and EDTA (**C**). Bar length is 50 nm.

The native iron core of Dps-like proteins (in vivo) contains only tens of iron atoms [32], but typically in vitro an iron storage compartment can accommodate up to 500 iron atoms [16,32,33]. Each iron atom is coordinated with six ligands in an octahedral manner. Compared to magnesium, iron cations have a completely different effect on the Dps-DNA co-crystals. An addition of 20 mM FeSO_4_ to the Dps-DNA solution leads to a complete destruction of the complex, as evidenced by the absence of Bragg peaks on the scattering curve (Figure 3A, curve 2). Moreover, curve 2 displays a sharp upward of the scattering intensity in the interval of scattering vectors s < 0.45 nm^−1^, indicating presence of large aggregates. Generally, this scattering profile is characteristic for a highly polydisperse and disordered system. Cryo-EM image (Figure 3B) also demonstrates the destruction of the Dps-DNA complex and aggregation after addition of Fe cations to the solution, but a part of the Dps dodecamers remain free and stable, and some of them contain high contrast regions inside the inner cavity of the protein, which will be discussed later. These free Dps macromolecules are not detected by SAXS due to the strong scattering by large aggregates.

To study the possibility of restoration of Dps-DNA complex destroyed by Fe cations, 20 mM EDTA was added to the solution. As seen in Figure 3A, curve 3, the addition of EDTA led to even greater aggregation and sedimentation of the aggregates. However, cryo-EM images indicate that despite almost total aggregation of the main components of the solution (top and bottom parts of Figure 3C), the complex can be partially restored (central area of Figure 3C).

Moreover, it should be noted that the individual DNA molecules are clearly visible in Figure 3C. This means that EDTA partially freed the negatively charged phosphodiester backbones of DNA from interaction with iron cations, which led to the possibility of partial restoration of the complex since some amount of the non-aggregated protein macromolecules remained in the solution (Figure 3B). Noteworthily, the absence of free Dps macromolecules after the introduction of the chelating agent into the solution (Figure 3C) indicates that all the remaining Dps particles interacted with DNA, forming the complex.

A necessary step of this research was to analyze the effect of iron cations on the structure of the Dps protein.

**Figure 4:**
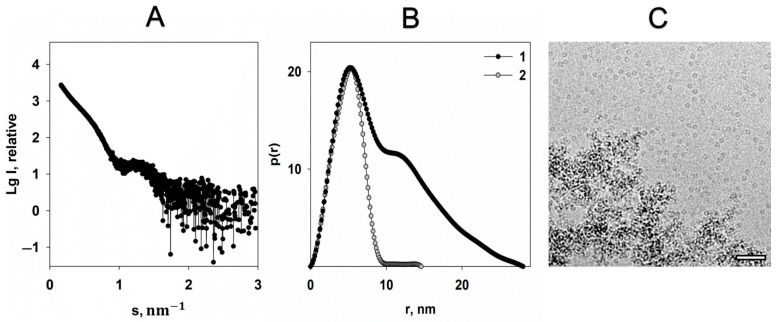
The effect of 20 mM FeSO_4_ on the Dps structure. (**A**) The experimental scattering curve of the Dps protein in the presence of 20 mM FeSO_4_ in solution (1). (**B**) The distance distribution functions *p(r)* of Dps in the presence of 20 mM FeSO_4_ (1) and Dps without FeSO_4_ (2). (**C**) CryoEM data of Dps with addition of FeSO_4_. Bar length is 50 nm.

The scattering curve obtained from Dps protein solution with addition of 20 mM FeSO_4_ (Figure 4A) indicates partial protein aggregation, expressed by the upturn of the scattering intensity in the region of scattering vectors s < 0.6 nm^−1^.

Nonetheless, the scattering from the individual Dps macromolecules is also present on the graph. A comparison of the distance distribution functions *p*(*r*) calculated using scattering curves of the pure protein and protein with addition of iron cations (Figure 4B) shows that *D_max_* becomes twice as large for the sample containing Fe^2+^, which is clear evidence of partial aggregation. However, location and profile of the first maxima of the *p*(*r*) functions coincide, demonstrating unchangeable size of the individual dodecamer. Cryo-EM data correlate well with SAXS results (Figure 4C).

Thus, both SAXS and cryo-EM data show that while the majority of Dps particles aggregate in the presence of iron cations, single Dps dodecamers also exist in solution. However, the question remains unanswered: Do iron atoms accumulate inside the dodecamer in this case?

### 2.3. Cryo-EM Single Particle Analysis of Dps with Accumulated Iron

**Figure 5:**
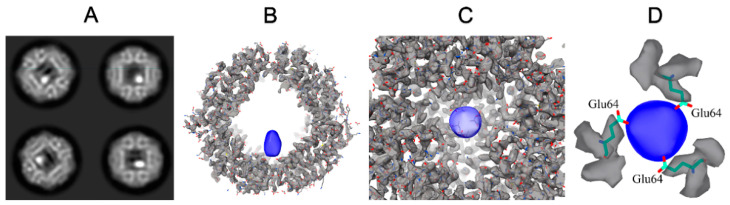
Dps-iron complex. (**A**) Example of 2D classification results; white dots correspond to iron clusters. (**B**–**D**) Cryo-EM map (EMD-12961); blue indicates the iron cluster and gray the Dps particle.

**Figure A1:**
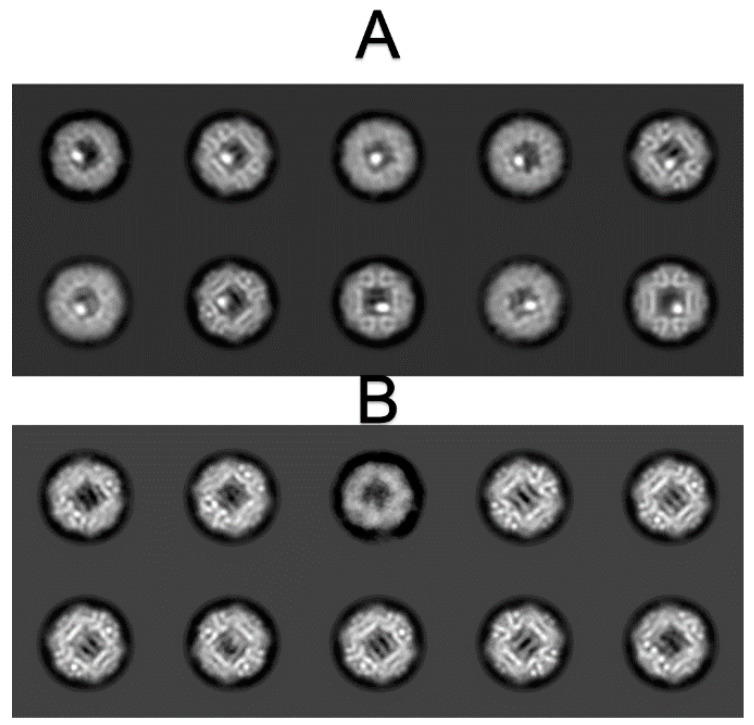
Tow-dimensional classification results of Dps protein with FeSO_4_. (**A**)—Dps particles with iron clusters; (**B**)—“empty” Dps particles.

Cryo-EM data demonstrate the presence of single Dps particles and Dps-containing aggregates. Two-dimensional classification revealed that 55% of the Dps dodecamers contain 10–15 Å wide high-contrast iron clusters inside the cavity (Figure 5A and Appendix A Figure A1A). Despite the P23 symmetry of the Dps dodecamer, all classes showed only one (or zero) iron cluster stored inside the cavity (Figure A1).

The 3.2 Å resolution density map (Figure 5B–D) obtained allows visualization of individual amino acidic residues. Although the iron clusters are elongated in one direction due to the reconstruction procedure (Figure 5B), the cryo-EM map unambiguously demonstrates that the iron clusters are stored inside the Dps cavity, close to the acidic (hydrophobic) pore and near the three glutamate residues (Glu64 on PDB 1DPS, Figure 5D). The cryo-EM map obtained was deposited in EMDB under the code EMD-12961.

## 3. Materials and Methods

### 3.1. Preparation of Dps and DNA Samples

The circular vector pcDNA-hIRR-GFP (9900 bp) was used as a DNA sample, which was prepared and isolated as described in [21,34]. After isolation, DNA was precipitated with isopropanol, washed with 70% ethanol, air dried and dissolved in a buffer containing 50 mM NaCl, 0.5 mM EDTA, 50 mM Tris-HCl pH 8.0 to a concentration of 3 mg/mL.

Overexpression and purification of *E. coli* Dps (UniProtKB-P0ABT2 (DPS_ECOLI)) was performed as described in [21,22]. The purified protein was concentrated on an Amicon ultrafiltration unit with a molecular weight cutoff of 10 kDa to a concentration of 3 mg/mL, and then dialyzed in a buffer of 50 mM NaCl, 0.5 mM EDTA, 50 mM Tris-HCl pH 8.0.

A buffer containing 50 mM NaCl, 0.5 mM EDTA, 50 mM Tris-HCl pH 8.0 was used for the preparation of Dps-DNA co-crystals, since it was shown to be most favorable for co-crystallization [21].

For preparation of Dps-DNA co-crystals, we used a Dps/DNA weight ratio of 5:1 according to the previous results [22].

To study the effect of metal ions, FeSO_4_ or MgCl_2_ salts were added to Dps or Dps-DNA solutions to a final concentration of 20 mM. Then, to remove metals and restore the original structures, EDTA was added to these solutions to a final concentration of 20 mM.

### 3.2. Solution Scattering Experiments and Data Analysis

Synchrotron SAXS measurements were performed at the European Molecular Biology Laboratory (EMBL) on the storage ring PETRA III (DESY, Hamburg) on the EMBL-P12 beamline equipped with a robotic sample changer and a 2D photon counting pixel X-ray detector Pilatus 6M (DECTRIS, Switzerland). The scattering intensity *I*(*s*) was recorded in the range of the momentum transfer 0.08 < *s* < 3.0 nm^−1^, where *s* = (4*πsin*2*θ*)/*λ*, 2*θ* is the scattering angle, and *λ* = 0.124 nm is the X-ray wavelength [35]. The measurements were carried out in the 50 mM NaCl, 0.5 mM EDTA, 50 mM Tris-HCl buffer, pH 8.0, at 10 °C using continuous flow operation over a total exposure time of 1 s, divided into 20 × 50 ms individual frames to monitor for potential radiation damage (no radiation effects were detected) [36]. The data were corrected for the solvent scattering and processed using standard procedures [37]. To account for the interparticle interactions, solutions of Dps at three concentrations in the range of 1–3 mg/mL were measured and the data extrapolated to infinite dilution using PRIMUS [23].

The distance distribution function *p*(*r*) was computed by the GNOM program [26] using the equation:

(1) $$p\left(r\right)=\frac{1}{2\pi^{2}}\overset{\infty }{\underset{0}{\int }}srI\left(s\right)\sin\left(\mathrm{sr}\right)ds\;$$

The maximum particle size (*D_max_*) was determined from the condition *p*(*r*) = 0 at *r* > *D_max_*. SAXS analysis and model fitting were performed in the most informative part of the range of the scattering curve 0.08 < s < 3.0 nm^−1^. The low-resolution shapes of the full-length Dps protein were reconstructed ab initio from the *p*(*r*) function by using an ab initio procedure and the DAMMIN program [27]. To minimize the discrepancy, the program utilizes a simulated annealing algorithm to build models fitting the experimental data *I_exp_*(*s*):

(2) $$\chi^{2}=\frac{1}{N-1}\underset{j}{\sum }{\left[\frac{I_{exp}\left(s_{j}\right)-cI_{calc}\left(s_{j}\right)}{\sigma \left(s_{j}\right)}\right]}^{2}$$

i.e., the reduced *χ*^2^ test, where *N* is the number of experimental points, *c* is a scaling factor and *I_calc_*(*s_j_*) and *σ*(*s_j_*) are the calculated intensity from the model and the experimental error of the momentum transfer *s_j_*, respectively. The reconstruction of the protein structure and the structures of flexible N-terminal fragments, which are absent in the crystallographic structure of the Dps protein, was performed using a hybrid method and the CORAL program [28], which combines an ab initio algorithm with rigid-body modeling. The program utilizes high-resolution structures from the Protein Data Bank and the theoretical intensities calculated by the CRYSOL program [38], and adds fragments of the macromolecule missing in the high-resolution model by using an ab initio procedure. The atomic-resolution structure of the Dps protein (PDB ID:1DPS) was used for the hybrid modeling.

The protein models reconstructed by different methods were analyzed with the SUPCOMB program [39] to determine the dissimilarity in their structural organization and find the normalized spatial discrepancies (NSD).

To analyze the volume fraction of free Dps and DNA in solution, the OLIGOMER program was used [23]. To determine the volume fractions *v_k_* of each component of the mixture, the program finds a linear combination of the scattering intensities from each component *I_k_*(*s*). In this case, the scattering intensity *I*(*s*) is described by the equation:

(3) $$I\left(s\right)=\overset{K}{\underset{k=1}{\sum }}\left(v_{k}I_{k}\left(s\right)\right)$$

OLIGOMER uses a non-negative linear least squares algorithm to minimize the *χ*^2^ discrepancy between the predicted mixture scattering curve and the experimental SAXS data.

### 3.3. Cryo-Electron Microscopy

Ten microliters of Dps solution was mixed with 15 µL of buffer solution (50 mM NaCl, 0.5 mM EDTA, 50 mM Tris-HCl pH 8.0); then 25 µL of DNA solution was added and the mixture was kept for 5 min at room temperature. For the studies of ion interaction, 50 µL of the Dps-DNA solution obtained or pure Dps was mixed with 1 µL of 1M MgCl_2_ or FeSO_4_. To study the effect of EDTA on complex structure, 1 µL of 1M EDTA was added to the solutions.

Three microliters of the Dps-DNA solution mixture obtained was applied to a Lacey EM grid (Ted Pella, USA) glow discharged for 30 s at 0.26 mbar pressure using current of 25 mA with Pelco EasiGlow (Ted Pella, Northport, NY, USA). The grids were then blotted with filter paper for 2.5 s from both sides at T = 20 °C and vitrified using Vitrobot Mark IV (ThermoFisher Scientific, Hillsboro, OR, USA).

Cryo-EM study was conducted using Titan Krios (ThermoFisher Scientific, Hillsboro, OR, USA) equipped with Falcon 2 direct electron detector (ThermoFisher Scientific, Hillsboro, OR, USA) and Image Corrector (CEOS, Germany) operated at 300 kV. Images were obtained using EPU software (ThermoFisher Scientific, Hillsboro, OR, USA) at 37,000× magnification with 1.72 Å pixel size, defocus ~3 µm and total dose ~50 *e*/Å^2^.

For single particle analysis (SPA), 129 movies of a Dps-FeSO_4_ sample were obtained at 75,000× with 0.86Å pixel size. Each movie consisted of 40 frames and was collected for 2 s. Total dose per movie was ~80 *e*/Å^2^*;* defocus values were in the range of 0.8–2.0 µm. Drift correction, CTF and defocus estimation, particle picking and extraction were conducted with Warp [40]; 77,769 particles were chosen for further processing in CryoSPARC [41] and Relion3 [42].

After initial rounds of 2D classification 49,861 Dps dodecamers were chosen for further processing. “Empty” Dps particles (2D classes shown in Figure A1) were utilized for ab initio model building, which was later used for the reconstruction of Dps with iron clusters. Since 2D classification demonstrated the preferred orientation of Dps particles (Figure 5A), tetrahedral (P23) symmetry was used to compensate for it, therefore three extra densities corresponding to iron clusters formed on the obtained cryo-EM maps and were manually erased later. After 3D classification, 11,215 particles were chosen for final reconstruction (Figure 5B–D, EMD-12961). The Dps density map is shown after local sharpening. Visualization threshold for iron clustering (Figure 5B–D) was chosen in accordance with the size of the cluster on 2D classes (Figure 5A).

## 4. Conclusions

This study was necessary not only for understanding of the process of formation of the protective bacterial complex, but also to find a possibility to control it. Divalent metal cations play a significant role in this phenomenon. Various divalent metals, including copper and zinc, are important for the interaction of nucleotides with proteins [25], but magnesium and iron are of special importance for the Dps-DNA protective complex. First, it was suggested that dodecameric Dps cannot directly bind DNA, and that the complex formation with DNA is mediated through ion bridges formed by Mg^2+^ [8]. Second, the Dps protein protects bacterial cells performing dual functions: (1) DNA binding and (2) iron oxidation and accumulation. Since Dps is a key component of the complex, on which the efficiency of bacterial protection depends, we investigated the effect of metal cations not only on the structure of the already formed complex, but also on the single Dps molecules.

Therefore, here we demonstrate the effect of 20 mM Mg^2+^ and Fe^2+^ ions on the structure of the Dps protein and on the Dps-DNA co-crystals. It was shown that the addition of a given amount of magnesium and iron cations leads to the destruction of the co-crystals, but the results of their action are different: interaction with Mg^2+^ leads to the reversible complex disintegration, while a presence of iron cations causes a total aggregation and only an excess of Dps molecules can lead to partial restoration of the complex. This can be explained by the differing nature of these metals. The main difference is that iron is a six-bond transition metal, while magnesium is just divalent. Iron can form stable complexes with nitrogen of different amino groups of the protein residues, which in turn can lead to the aggregation of neighboring protein molecules.

Analysis of the effect of the divalent ions on the structure of the Dps protein showed that the addition of MgCl_2_ brings N-terminals closer to the Dps surface, making them inaccessible for binding with DNA. At the same time, the addition of FeSO_4_ leads to a practically total aggregation, but also to the formation of iron-containing clusters in the central cavity of the protein. A three-dimensional reconstruction of the Dps structure with an iron-containing cluster was carried out with a spatial resolution of 3.2 Å. It was shown that an iron-containing cluster with a 10–15 Å diameter, which corresponds to the several tens of the iron atoms, is located in the cavity of the Dps dodecamer next to the hydrophobic pore, near three glutamic acids.

## Data Availability

All data are available in the manuscript, electron microscopy density maps are deposited in the EMDB (EMD-12961).
